# Oral Granulomatous Disorders: A Diagnostic Insight

**DOI:** 10.7759/cureus.65742

**Published:** 2024-07-30

**Authors:** Durba Roychowdhury, Rudra Prasad Chatterjee, Swagata Gayen, Sanjeet Das, Arunit Chatterjee, Sudeshna Bagchi, Mousumi Pal, Rhitam Ghosal, Anwesha Paul, Shreya Batabyal

**Affiliations:** 1 Oral and Maxillofacial Pathology, Guru Nanak Institute of Dental Sciences & Research, Kolkata, IND

**Keywords:** caseation necrosis, non-caseating, management, inflammation, granuloma

## Abstract

Granulomatous inflammation represents a unique pattern of chronic inflammation observed in a restricted form of infectious and certain non-infectious diseases. The formation of granulomas typically involves immune responses. Granulomatous disorders encompass a broad spectrum of conditions that share the common histological feature of granuloma formation. Their involvement in the oral soft and hard tissues is quite infrequent; however, their manifestation can pose a diagnostic challenge due to the diverse range of potential causes and the relatively non-specific appearance of the individual lesions. The ultimate outcome of a complex entails the formation of a granuloma, resulting from the interplay among an invading pathogen or antigen, chemical substance, medication, or other irritant, persistent presence of antigens in the bloodstream, activation of macrophages, initiation of Th1 cell response, B-cell overactivity, presence of circulating immune complexes, and a wide range of biological signaling molecules, ultimately leading to the development of fibrosis attributed to the actions of transforming and platelet-derived growth factor. This article emphasizes the clinicopathological diagnostic criteria of oral granulomatous disorders as a guide for treatment and management.

## Introduction and background

The term "granuloma" originates from the word "granule," denoting a circumscribed granule-like lesion, and "-oma," a suffix frequently employed for benign growths; however, in this context, it signifies a localized inflammatory aggregation or accumulation of macrophages [1].

Robbins and Cotran defined granuloma as “a focus of chronic inflammation consisting of a microscopic aggregation of macrophages that are transformed into epithelium-like cells, surrounded by a collar of mononuclear leukocytes, principally lymphocytes and occasionally plasma cells” [2].

Granulomatous diseases encompass a vast array of conditions that are characterized by the common histological features of granuloma development [3].

Granulomatous disorders have afflicted human populations for millennia, as indicated by the presence of tuberculosis infection in ancient Egyptian mummies. The description of syphilis has been attributed to Hippocrates, and it was identified as a venereal disease in the 15th century. By the 17th century, observations were made of tiny granules (miliary) in the tissues of hosts [4].

Granulomas consist predominantly of macrophages, especially epithelioid histiocytes, frequently accompanied by multinucleated giant cells along with varying numbers of CD4+ T lymphocytes. The polarization of macrophages holds significance in the development of granulomatous disorders. These macrophages can be categorized into two variants: M1 and M2. M1 macrophages which appear in the early stages of the disease, undergo classical activation through cytokines, like interleukin-2, interferon-γ, and tumor necrosis factor (TNF)-α, primarily engaging in the eradication of microbes. Conversely, M2 macrophages (also known as alternatively activated macrophages) often emerge later or during the resolution of the disease, expressing anti-inflammatory cytokines, and mainly contributing to immunoregulation and tissue modeling, including fibrosis. Consequently, they are regarded as a cellular hallmark of chronic inflammation [5,6,7,8].

The granulomatous inflammatory response is widespread in pathology, manifesting in a variety of infectious, toxic, allergic, autoimmune, and neoplastic disorders, as well as conditions of unknown etiology [9].

Granulomatous inflammation of the oral soft and hard tissues is a rare phenomenon, but when identified, the diversity of potential causative diseases and the somewhat generic appearance of the individual lesions provide an unambiguous diagnostic challenge [4].

Taking this discussion into account, this article accentuates the current knowledge about the etiology, classification, clinicopathological diagnosis, and prognostic features of granulomatous disorders affecting the orofacial region.

## Review

Granulomatous inflammation

Granulomatous inflammation represents a characteristic pattern of chronic inflammation observed in a limited array of infectious and some non-infectious conditions. Typically, granulomas develop as a consequence of immune reactions [2].

Pathogenesis of Granuloma

The progression of granuloma formation is depicted in a schematic manner and is succinctly described below:

The granuloma is a functional site of various cytokines and enzymes, as well as fibronectin and multiple progression factors that contribute to aging. Macrophages and monocytes phagocytose the antigen in an attempt to eliminate it. However, due to the antigen's low degradability, these cells are unable to effectively break it down, leading to a transformation into epithelioid cells. As antigen-presenting cells, macrophages, having been unsuccessful in addressing the antigen, proceed to present it to CD4+T lymphocytes with increased expression of MHC class II molecules. Physiological stimulation of histiocytes takes place within a period of 24 to 48 hours following an injury through the activation of various components, such as complement (C3b, C5a), helper T cells (Th1) that secrete chemokines, and cytokines (TNF, IL-1, IL-6, IL-17, and IFN-γ). These substances play a crucial role in stimulating, attracting, and guiding macrophages toward the specific location of the injury [1,10,11,12].

Interferon-gamma (IFN-γ) is responsible for the activation of these macrophages by enhancing MHC class II molecules, and activated macrophage receptors carry an Fc component of IgG to intensify their capability for phagocytosis [1,3].

**Figure 1:**
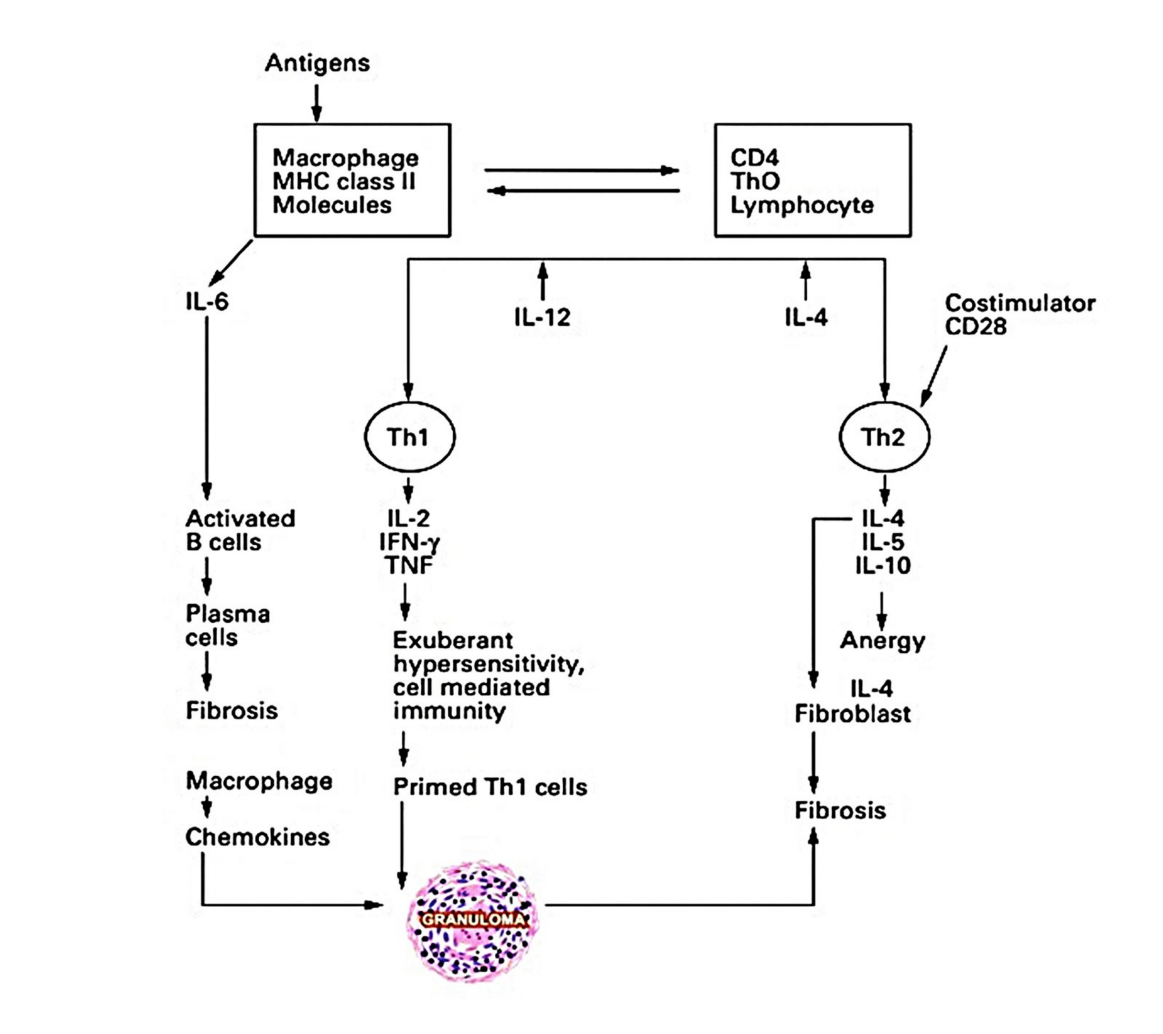
Pathogenesis of a granuloma. IL: interleukin, IFN-γ: interferon-γ, TNF: tumor necrosis factor; MHC: major histocompatibility complex Permission has been obtained from the original publishers to reproduce this figure from the source [3].

The end result of a complex is the granuloma interaction between an invading organism or antigen, chemical, drug or other irritant, prolonged antigenemia, macrophage activity, a Th1 cell response, B-cell overactivity, circulating immune complexes, and a vast array of biological mediators and eventually develops into fibrosis due to transforming and platelet-derived growth factor (Figure 1) [3].

Areas of inflammation or immunological reactivity attract monocyte macrophages, which may merge to produce multinucleated giant cells and transform the macrophages into epithelioid cells [3].

The B7:CD28/CTLA:4 costimulatory pathway is also required by T-cell activation. CD28-mediated co-stimulator promotes active T-cell proliferation; without it, ignorance, anergy, and apoptosis can occur [3].

When Th1 cells are overstimulated in contrast to Th2 cells, significant cell-mediated hyperactivity, tissue damage, and granuloma formation ensue. B7-1 or B7-2 antagonists inhibit this process. The reverse scenario occurs when Th2 appears to overturn Th1 influences. Anergy and apoptosis can be reversed by using CD28 antagonists [3].

One would presume that the principal role of the granuloma is to sequester and, if feasible, eliminate an offending substance [3].

Composition of Granuloma

**Figure 2:**
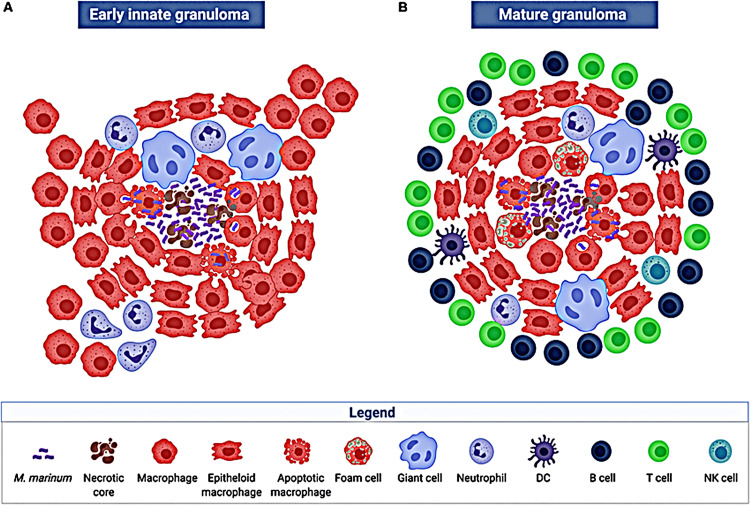
Structure and cellular composition of granulomas. Permission has been obtained from the original publishers to reproduce this figure from the source [13].

In general, granuloma comprises the components shown in Figure 2. 

Epithelioid cells: These are so named because they resemble epithelial cells, which are modified macrophages/histiocytes with elongated cells and a slipper-shaped nucleus. These cells exhibit vesicular, light-staining nuclear chromatin and abundant, pale-staining cytoplasm with fuzzy outlines, resulting in the intimate apposition of the cell membranes with adjacent epithelioid cells which have a low phagocytic activity [1].

Multinucleated giant cells: Adjacent epithelioid cells fuse to form multinucleated giant cells and may have 20 or more nuclei, which may be organized at the periphery, like the horseshoe or as a ring, or may be crowded at the two poles (Langhans’ giant cells), or they may be located centrally (foreign body giant cells). The former are commonly seen in tuberculosis. While the foreign body tissue reactions are typical in the latter, tuberculosis is common in the former. These giant cells have a low phagocytic activity similar to the epithelioid cells, but they do secrete compounds that aid in eliminating the invasive agents [1].

Lymphoid cells: The lymphocyte host response is an essential part of granuloma composition, functioning as a cell-mediated immunological reaction to antigen. There are several forms of granulomas containing plasma cells that indicate an increased humoral immune response [1].

Necrosis: A couple of granulomatous diseases, such as central caseation necrosis in tuberculosis, so named because of its dry, cheese-like appearance, may exhibit necrosis [1].

Fibrosis: Fibrosis occurs when fibroblasts proliferate at the edges of a granuloma during healing [1].

The quintessential illustration of granulomatous inflammation involves the tissue reaction to tubercle bacilli, known as tubercle observed in cases of tuberculosis. A mature tubercle typically measures around 1 mm in diameter, featuring a central region of caseation necrosis, encircled by epithelioid cells and one to multiple multinucleated giant cells (often of Langhans’ type), enveloped at the outer edge by lymphocytes and delimited by fibroblasts and fibrous connective tissue [1].

Granulomatous disorders

Granulomatous disorders can be caused by infections, vasculitis, immunological disorders, hypersensitivity, neutrophil oxidase defects, certain chemicals, and neoplasia [9]. Caseating and non-caseating granulomas represent distinct forms of inflammatory lesions. The former is distinguished by the presence of deceased or necrotic material with a consistency reminiscent of cheese, known as “caseous necrosis,” which comprises eosinophilic remnants devoid of nuclear or cytoplasmic outlines. By contrast, non-caseating granulomas lack necrotic components and are comprised of activated immune cells like macrophages and T-lymphocytes [14].

Classification

Based on the type of necrosis and etiology, the classification of oral granulomatous diseases [3,4,15,16] is mentioned in Table 1 and Table 2.

**Table 1:** Classification of oral granulomatous diseases based on the type of necrosis. Permission has been obtained from the original publishers to reproduce this table from the source [15].

Type of necrosis	Granulomatous lesions
Caseating granulomas/suppurative	Tuberculosis, syphilis, cat scratch disease, actinomycosis, blastomycosis, cryptococcosis, coccidioidomycosis
Non-caseating granulomas/non-suppurative	Leprosy, sarcoidosis, Crohn’s disease, silicosis, foreign body granulomas

**Table 2:** Classification based on the etiology. Permission has been obtained from the original publishers to reproduce this table from the source [3,4,15,16].

Si. No.	Etiology	Name of the disease
I. Infectious diseases		
A.	Bacterial	a) Tuberculosis
		b) Leprosy
		c) Syphilis
		d) Brucellosis
		e) Actinomycosis
		f) Cat scratch Disease
		g) Anthrax
B.	Fungal	a) Histoplasmosis
		b) Blastomycosis
		c) Phycomycosis (mucormycosis)
		d) Aspergillosis
		e) Coccidioidomycosis
		f) Rhinosporidiosis
		g) Paracoccidioidomycosis
		h) Cryptococcosis
		i) Candidiasis
C.	Parasitic	a) Leishmaniasis
		b) Myiasis
		c) Toxoplasmosis
D.	Spirochaetes	a) *T.* *pallidum*
		b) *T. carateum *
		c) *T. pertenue *
II.	Traumatic	a) Pyogenic granuloma
		b) Reparative granuloma
III.	Foreign body	a) Oral foreign body reactions (suture, amalgam, endodontic sealer, etc.)
		b) Cholesterol granuloma
		c) Cocaine-induced midline granuloma
		d) Gout
IV.	Neoplastic	a) Histiocytosis X-
		i) Eosinophilic granuloma
		ii) Hand-Schüller-Christian disease
		iii) Letter-Siwe disease
		b) Benign fibrous histiocytoma
		c) Necrotizing sialometaplasia
		d) Polymorphic reticulosis (lethal midline granuloma)
V.	Others (unknown, autoimmune, & vascular)	a) Sarcoidosis
		b) Crohn’s disease
		c) Cheilitis granulomatosa
		d) Eosinophilic granuloma
		e) Wegener’s granulomatosis
		f) Sjogren’s syndrome
VI.	Developmental	a) Melkersson-Rosenthal syndrome
VII.	Miscellaneous	Congenital chronic granulomatous disease of childhood

A comprehensive classification of granulomatous disorders of the skin (according to the American Academy of Dermatology, 76th Annual Meeting San Diego, CA USA) is depicted in Table 3 [17,18].

**Table 3:** Classification of granulomatous disorders of the skin. Permission has been obtained from the original publishers to reproduce this table from the source [17,18].

Noninfectious granulomatous disorders	Infectious granulomatous disorders
Epitheliod granulomas	Caseating granulomas
Sarcoidosis	Tuberculosis
Granulomatous rosacea/ POD	Leprosy
Cutaneous Crohn’s	Atypical mycobacteria
Orofacial granulomas	Leishmaniasis
Palisading granulomas	Suppurative granulomas
Granuloma annulare	Deep fungal
Elastolytic giant cell granuloma	Pyodermas
Necrobiosis lipoidica	Granulomatous STDs
Rheumatic nodules	
Reactive granulomatous disorders	
Xanthomatous granulomas	
Adult-onset XG	
Adult-onset APXG	
NXG	
Multicentric reticulohistiocytosis	
Rosai-Dorfman	
Xanthoma disseminatum	
Others	
Granulomatous vasculitis	
Foreign body reactions	
Lymphomatoid granulomatosis	
Granulomatous drug reactions	

Clinicopathological Diagnostic Criteria of Granulomatous Disorders

Tuberculosis (TB): The oral manifestations of TB are as follows: Primary tuberculosis is reported in the gingiva, mucobuccal fold, and areas of inflammation adjacent to teeth or in extraction sites and presents as diffuse, hyperemic, nodular, or papillary proliferation of the gingival tissues and frequently associated with regional lymphadenopathy [19].

In secondary tuberculosis, the tongue is the most commonly affected site, followed by the palate, lip, buccal mucosa, and gingiva. It appears as an irregular, superficial, or deep, painful ulcer that tends to increase slowly in size. Occasional mucosal lesions show swelling and granular, nodular, or fissured lesions, but no obvious clinical ulceration. TB may also affect the maxilla or mandible. Tuberculous osteomyelitis manifests in the jaws, presenting as indistinct radiolucent areas. Extrapulmonary sites frequently encountered in the head and neck region are the cervical lymph nodes succeeded by the larynx and middle ear. Less prevalent sites encompass the nasal cavity, nasopharynx, oral cavity, parotid gland, esophagus, and spine [19,20].

**Figure 3:**
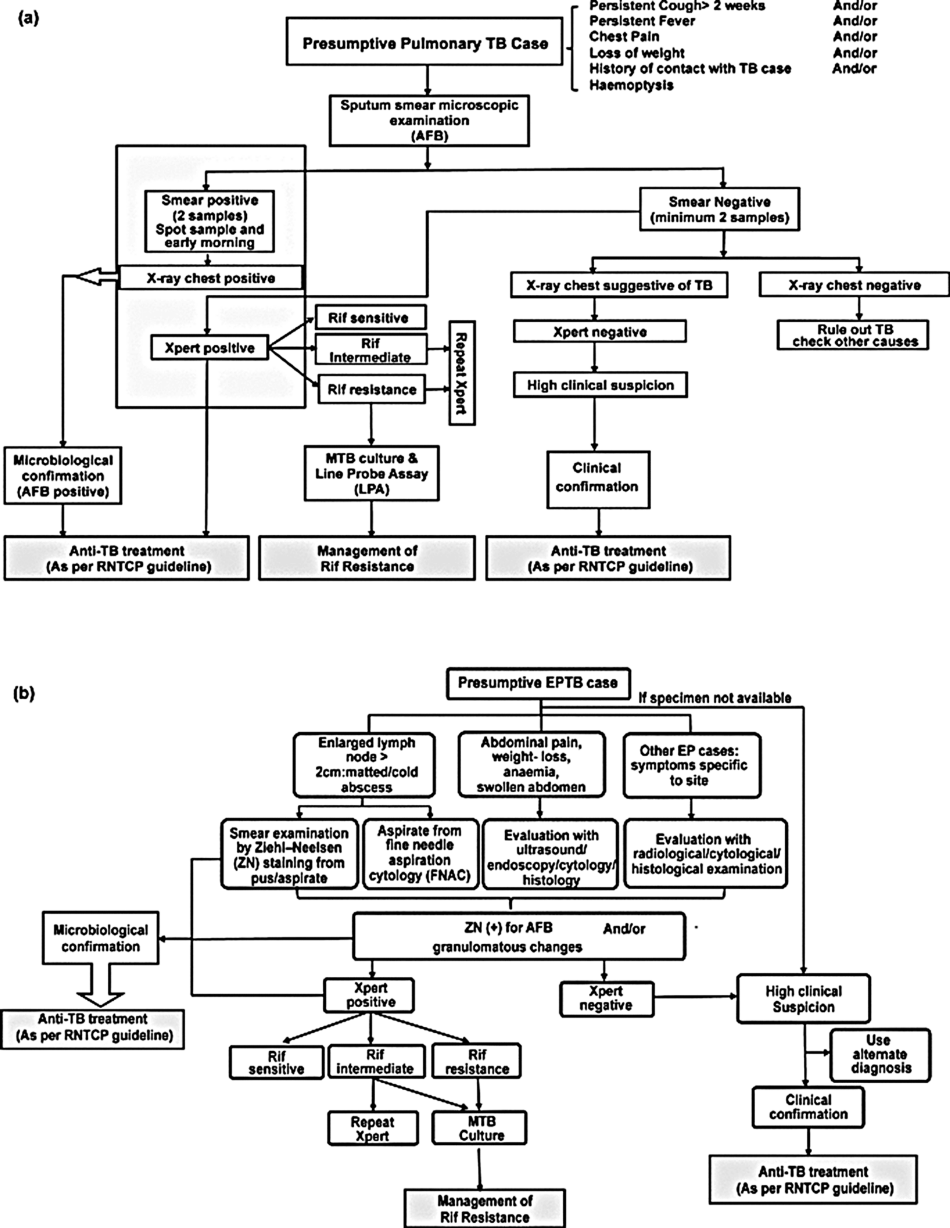
Diagnostic algorithm for a) pulmonary tuberculosis (TB) and b) extra-pulmonary tuberculosis (EPTB) as per the Revised National TB Control Program (RNTCP) guidelines. TB: tuberculosis, AFB: acid-fast bacilli, RIF: rifampicin Permission has been obtained from the original publishers to reproduce this figure from the source [22].

In India, the diagnosis of tuberculosis is conducted in accordance with the guidelines of the Revised National Tuberculosis Control Program, as shown in Figure 3a-3b, which aligns with the recommendations set forth by the World Health Organization [21].

**Figure 4:**
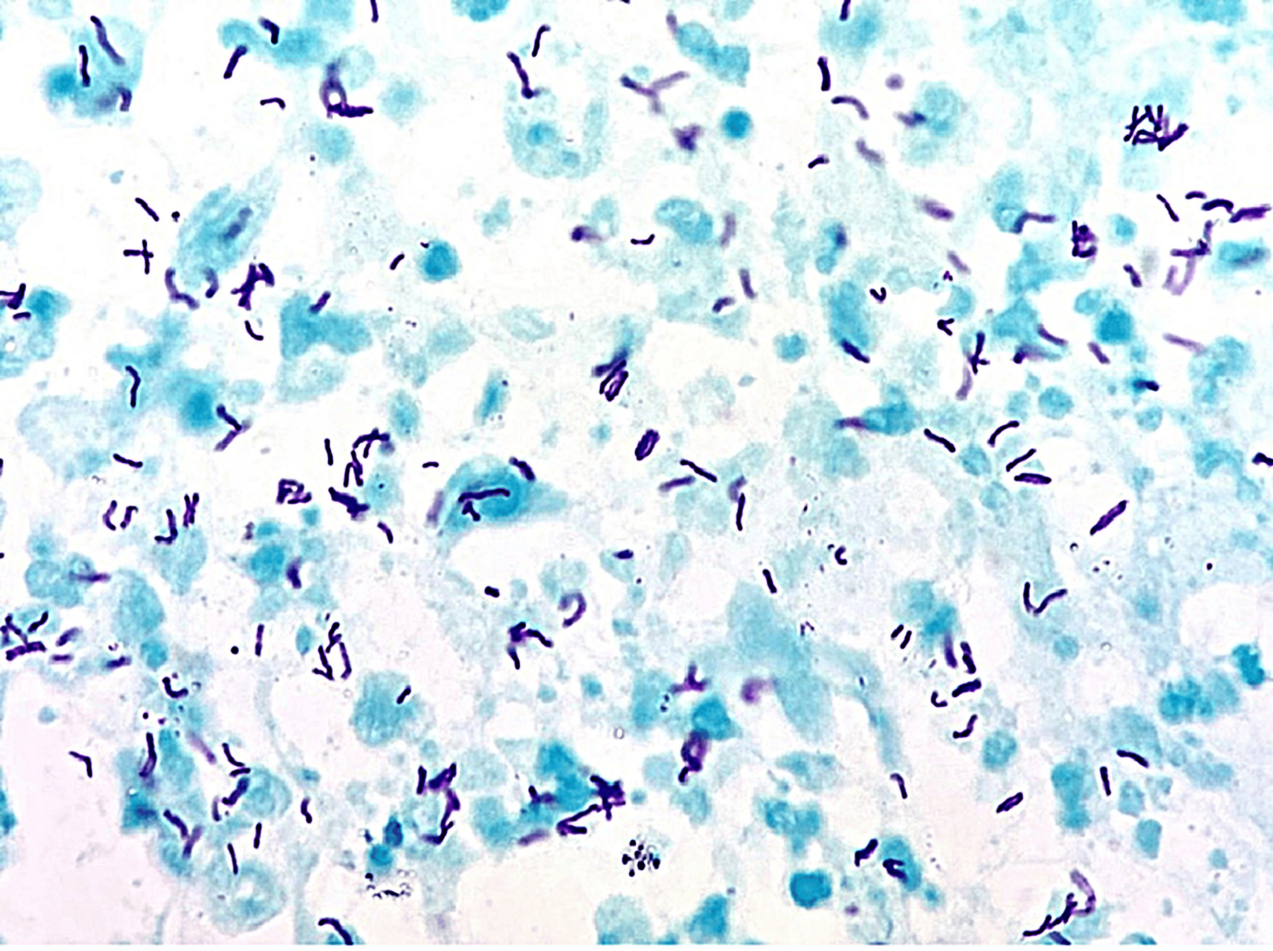
Positive acid-fast bacilli (AFB) in sputum smear. Permission has been obtained from the original publishers to reproduce this figure from the source [23].

Currently, while the diagnosis of pulmonary TB (PTB) through sputum-smear direct microscopy is relatively well-established, the identification of smear-negative PTB and extra-PTB (EPTB) presents significant obstacles due to the paucibacillary nature of the specimen (Figure 4).

The diagnosis of EPTB typically necessitates the collection of samples from the affected anatomical site through invasive and discomforting procedures that demand clinical proficiency. The introduction of Xpert MTB/RIF (Xpert), an automated molecular detection system capable of simultaneously identifying tuberculosis and rifampicin resistance, has revolutionized the diagnostic landscape [21,22].

**Figure 5:**
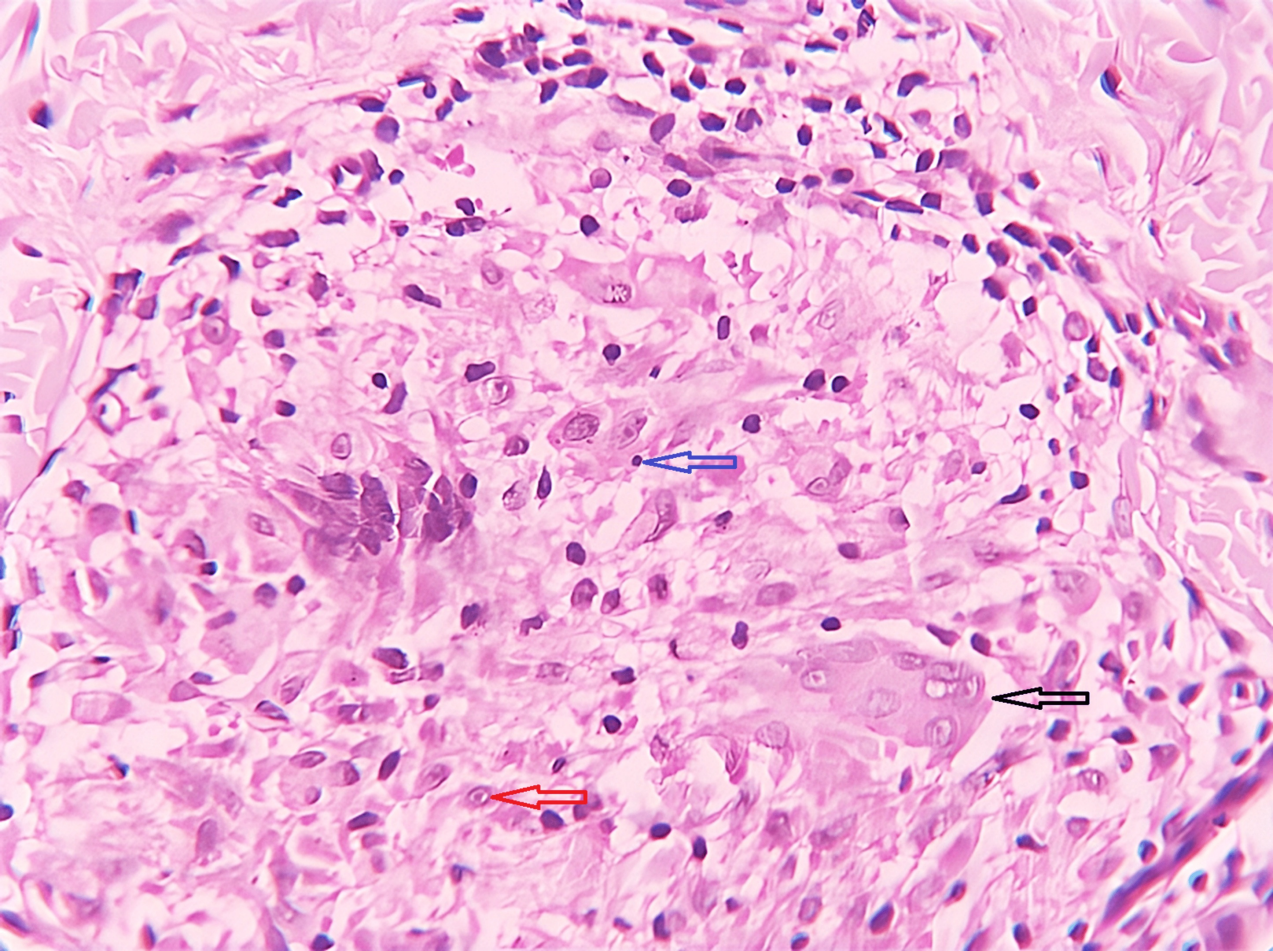
Oral tuberculosis: photomicrograph revealing tuberculous granulomas along with Langhans giant cells (black arrow), epithelioid cells (red arrow), and lymphocytes (blue arrow) (x40). Courtesy: College of Medicine & Sagore Dutta Hospital

The distinctive histopathological presentation is attributed to the immune-mediated hypersensitivity response. The fundamental microscopic change observed in TB is characterized by granulomatous inflammation, which is primarily composed of lymphocytes, epithelioid histiocytes, and multinucleated giant cells along with central caseous necrosis (75.5%) (Figure 5) [20,24].

Within tissues, *M. tuberculosis* triggers a distinct macrophage reaction, leading to the formation of localized areas where macrophages are encircled by lymphocytes and fibroblasts. These macrophages develop a copious eosinophilic cytoplasm, imparting a superficial resemblance to epithelial cells; hence, they are commonly referred to as epithelioid cells. The fusion of macrophages gives rise to the formation of Langerhans giant cells, characterized by nuclei arranged peripherally around the cytoplasm. With the progression of granulomas, central necrosis ensues, commonly termed caseous necrosis due to the coarse, cheesy consistency of these regions [25].

Leprosy (Hansen’s disease): This condition is most commonly found in the hard palate, soft palate, labial maxillary gingiva, tongue, lips, buccal maxillary gingiva, labial mandibular gingiva, and buccal mucosa. Small tumor-like masses, called lepromas, develop on the tongue, lips, or hard palate. This condition also presents with gingival hyperplasia with loosening of the teeth. They present as yellowish to red, sessile, firm, enlarging papules that undergo ulceration and necrosis, subsequently followed by healing through secondary intention. Prolonged infection of a specific area can result in significant scarring and tissue loss. Fixation of the soft palate and complete loss of the uvula may manifest. The lingual lesions appear mainly in the anterior third and typically originate as erosive areas, potentially evolving into large nodules. Lip infections can lead to macrocheilia; paralysis of the facial and maxillary division of trigeminal nerve; circumferential hypoplasia; shortening of roots, usually involving maxillary anterior teeth; long-standing lepromatous lesionsp; granulomatous invasion of the pulp; and pinkish discoloration of crowns [19,20].

**Figure 6:**
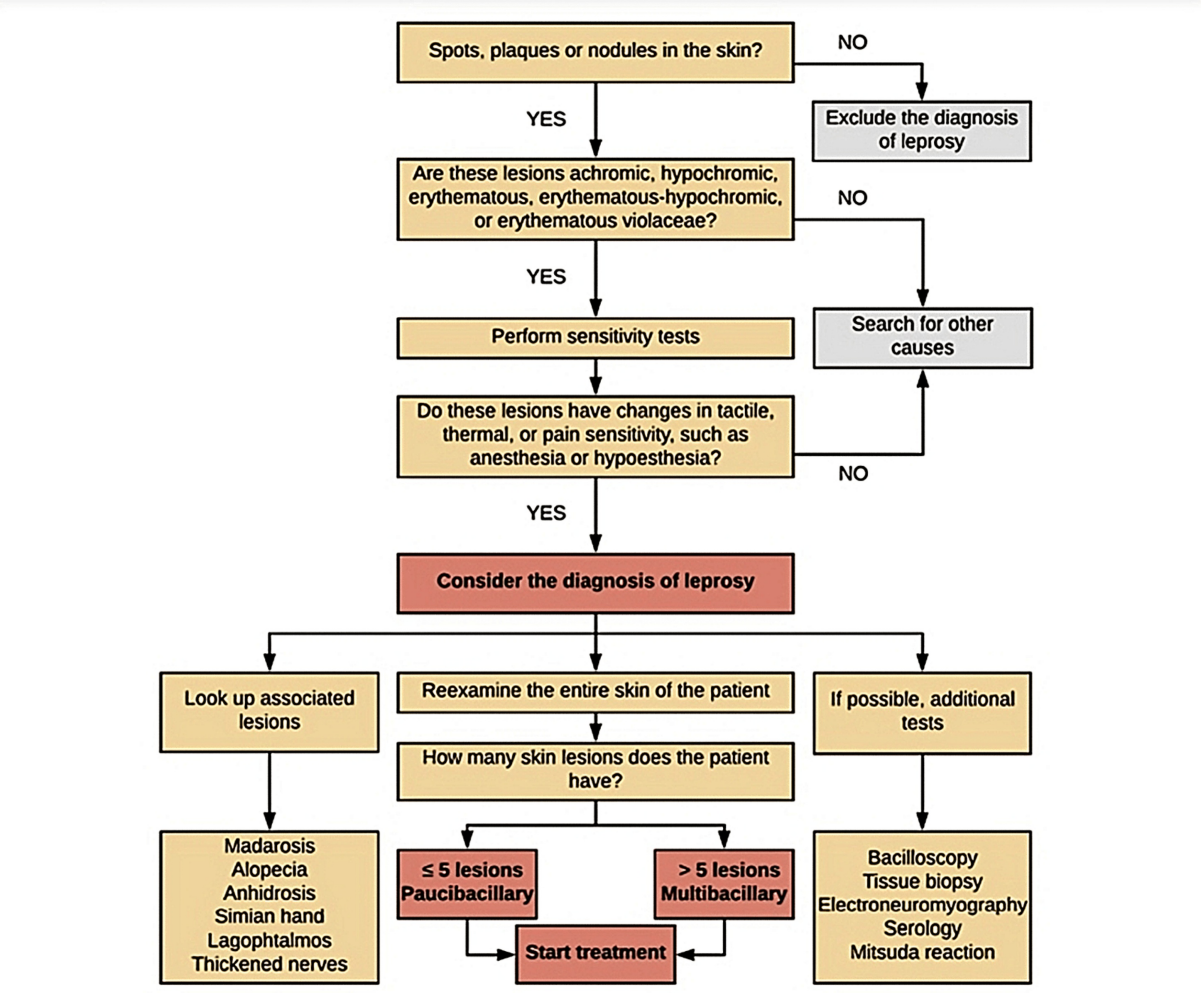
Schematic illustration of the approach to the patient with suspected leprosy according to the WHO criteria. Permission has been obtained from the original publishers to reproduce this figure from the source [27].

The introduction of a novel adjunctive examination could potentially influence various aspects of existing diagnostic protocols (Figure 6) [26].

**Figure 7:**
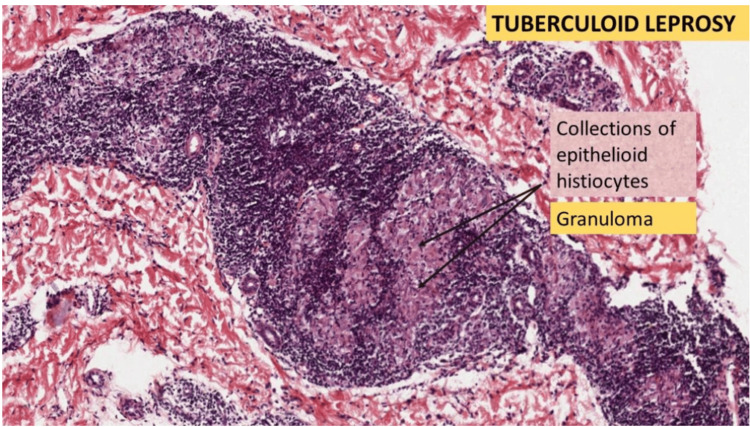
Paucibacillary (tuberculoid) leprosy. Well-formed granulomatou inflammation illustrating clusters of lymphocytes and histiocytes. Permission has been obtained from the original publishers to reproduce this figure from the source [29].

Biopsy samples from individuals with paucibacillary leprosy commonly exhibit the tuberculoid pattern, showcasing granulomatous inflammation characterized by well-organized clusters of epithelioid histiocytes, multinucleated giant cells, and lymphocytes; the infiltrate commonly exhibits a "sausage shape" as it progresses along the pathways of nerves and appendages [20,28]. The presence of organisms is minimal, and if detected, they can be demonstrated only with acid-fast dyes like the Fite method [20] [Figure 7].

**Figure 8:**
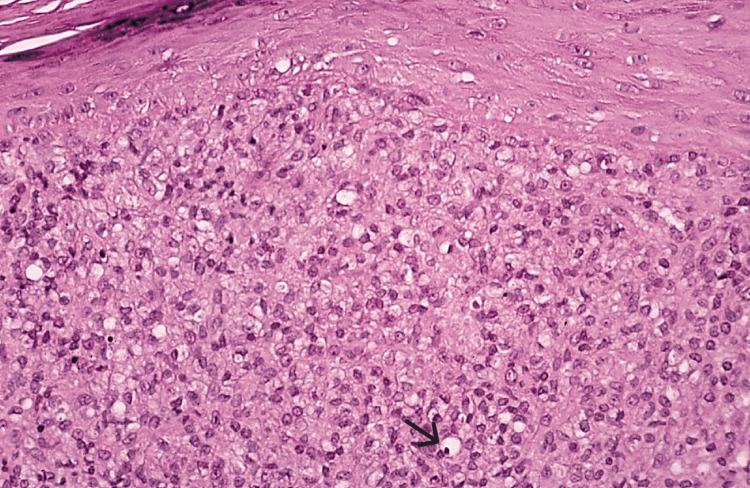
Multibacillary (lepromatous) leprosy. Sheets of lymphocytes and histiocytes displaying dispersed vacuolated lepra cells (black arrow). Permission has been obtained from the original publishers to reproduce this figure from the source [20].

Multibacillary leprosy (lepromatous) is characterized by a lepromatous presentation lacking well-defined granulomas wherein sheets of lymphocytes intertwined with vacuolated histiocytes forming lepra cells that are scattered throughout the lesion (Figure 8) [20].

**Figure 9:**
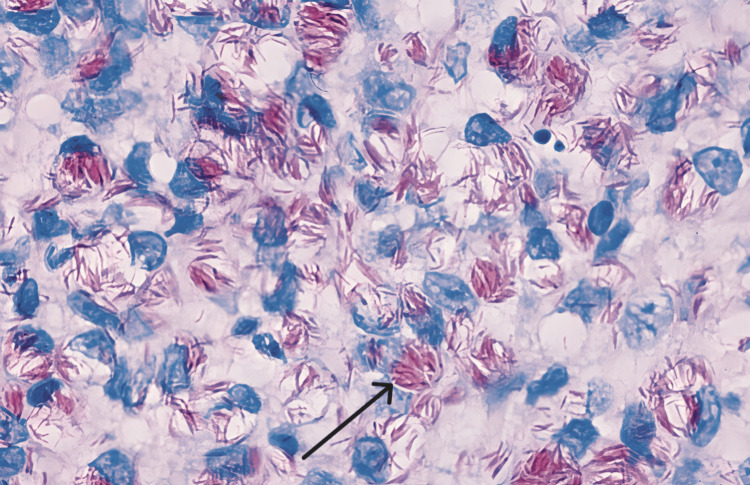
Multibacillary (lepromatous) leprosy. Acid-fast stain demonstrating numerous small mycobacterial organisms(black arrow) observed individually or in clusters. Permission has been obtained from the original publishers to reproduce this figure from the source [20].

In contrast to tuberculoid leprosy, the lepromatous variant exhibits a high concentration of organisms that are detectable using acid-fast staining techniques (Figure 9) [25].

Other investigations include skin biopsy, nerve biopsy, and foot culture histamine test. Tests for humoral response include monoclonal antibodies, ELISA, and PCR, among others. In children, the sweat function test was used [19]. 

Actinomycosis: Actinomyces may penetrate through the oral cavity and may either confine to the underlying soft tissues or spread to the salivary glands, tongue, occasionally gingiva, bone, or even the skin of the face and neck, producing swelling and induration of the tissue, which finally develops into one or more abscesses. They are more likely to discharge upon a skin surface, rarely a mucosal surface, liberating pus comprising of typical sulfur granules. The skin overlying the abscess is purplish red, indurated and feels woody, or often fluctuant. A new abscess forms and perforates the skin surface due to the chronicity of the disease. Scarring and disfigurement of the skin occur over a period of time. It may extend to involve the mandible or, very rarely, the maxilla, resulting in actinomycotic osteomyelitis, which may eventually involve the cranium, meninges, or the brain itself if the bone of the maxilla is invaded. Such destructive lesions within the bone may develop or confine at the apex of one or more teeth and simulate a periapical granuloma or cyst [19].

**Figure 10:**
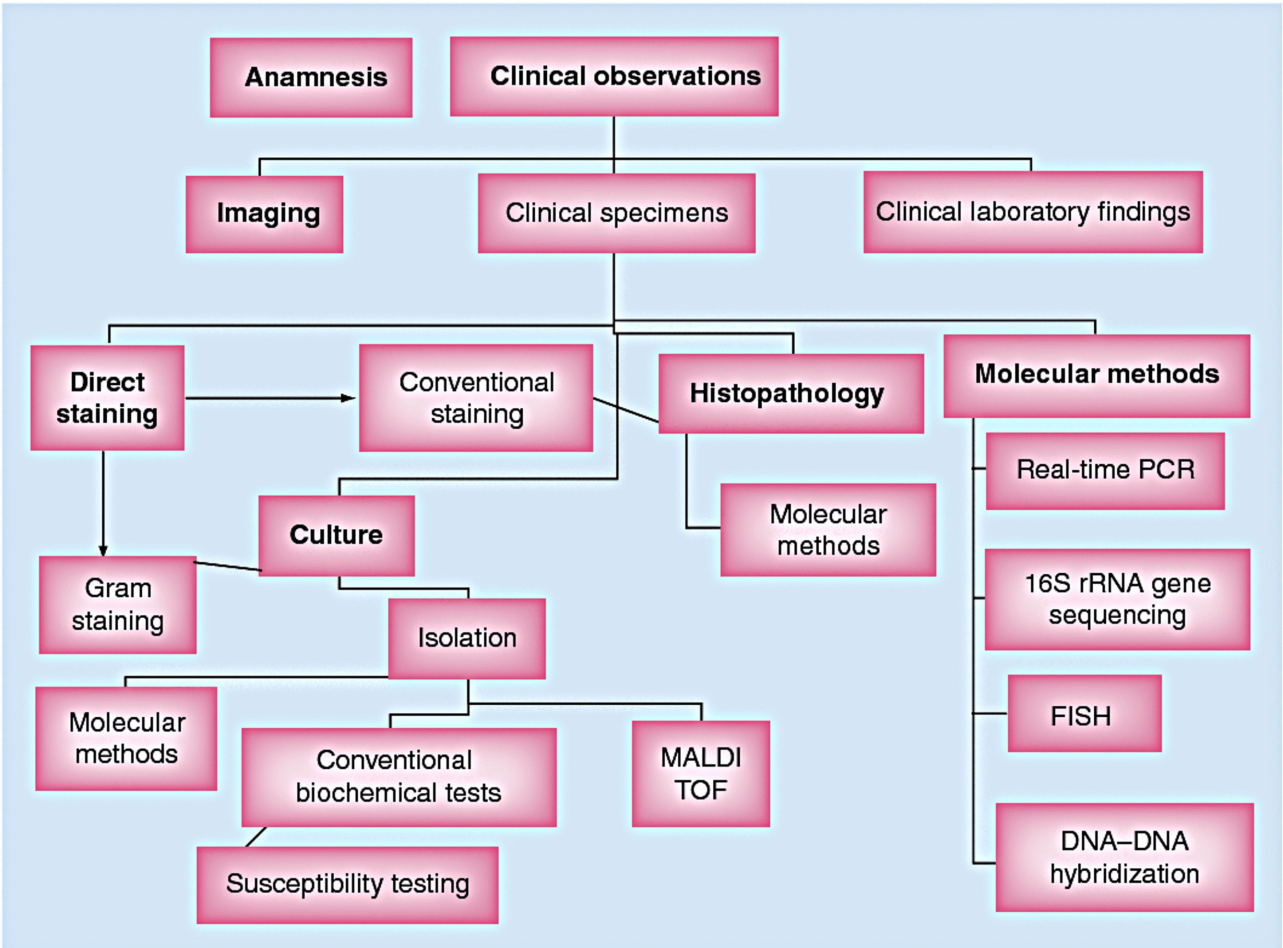
Diagnostic algorithm for actinomycosis. MALDI-TOF: matrix-assisted laser desorption ionization time-of-flight mass spectrometry Permission has been obtained from the original publishers to reproduce this figure from the source [31].

The process of microbiological identification in cases of actinomycosis involves isolating the responsible pathogen from a sterile anatomical site. Optimal clinical samples typically include deep needle aspirates, purulent material, sulfur granules extracted from draining sinuses, and tissue biopsy specimens (Figure 10) [30].

**Figure 11:**
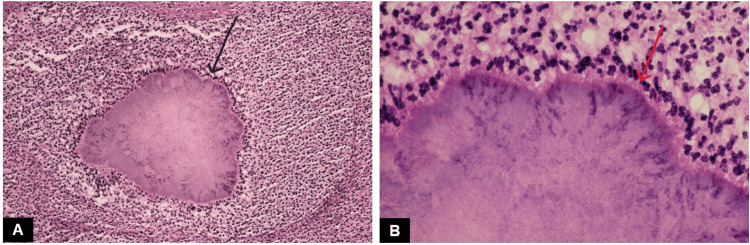
(a) Colony of actinomycotic organisms (black arrow) surrounded by polymorphonuclear leukocytes (x10). (b) Actinomycotic colony showing club-shaped filaments arranged in a radiating rosette pattern (red arrow) (x40). Permission has been obtained from the original publishers to reproduce this figure from the source [20].

Characteristic colonies of microorganisms surrounded by polymorphonuclear leukocytes are often associated with multinucleated giant cells and macrophages with peripheral fibrosis. The peripheral portion of the lesion showed eosinophilia while the core was stained with hematoxylin. Peripheral club-shaped ends of the filaments forming a radiating rosette pattern were observed. This unusual appearance of the colonies with the peripheral radiating filaments is the basis for the classically-used term "ray fungus." Methenamine silver stain is more effective in demonstrating the organisms [19,32] (Figure 11A, 11B).

**Figure 12:**
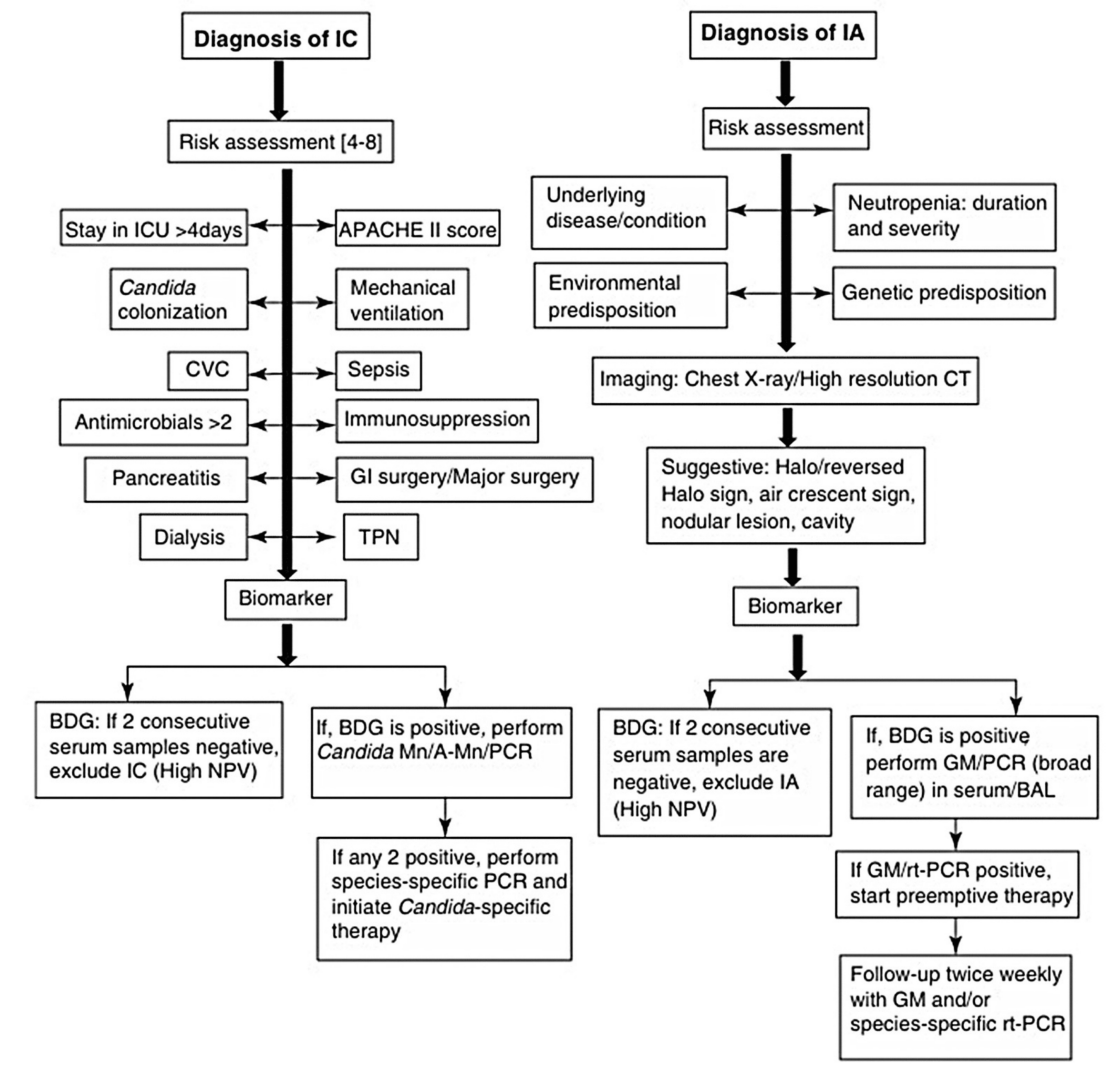
A step-by-step process for the diagnosis of invasive candidiasis (IC) and invasive aspergillosis (IA). CVC: central venous catheter, TPN: total parenteral nutrition, BDG: β-d-glucan, GM: galactomannan, rt-PCR: real-time PCR, Mn: Mannan, A-Mn: anti-Mannan antibody, NPV: negative predictive value Permission has been obtained from the original publishers to reproduce this figure from the source [33].

Fungal diseases: Biomarker-guided methodologies have currently evolved into customary practices aimed at facilitating prompt and precise identification of invasive fungal infections (IFIs) and have been incorporated within the updated European Organization for Research and Treatment of Cancer Mycoses Study Group (EORTC/MSG) consensual descriptions of invasive fungal diseases (IFDs). A pragmatic methodology for discerning invasive candidiasis (IC) and invasive aspergillosis (IA) is delineated in Figure 12 [33].

Histoplasmosis: Most oral lesions develop with the disseminated form of the disease. The tongue, palate, and buccal mucosa are the most commonly affected sites. They present as a solitary, variably painful ulceration persisting for several weeks that have firm, rolled margins and may be indistinguishable clinically from a malignancy. However, some lesions may appear erythematous or white with an irregular surface [20].

**Figure 13:**
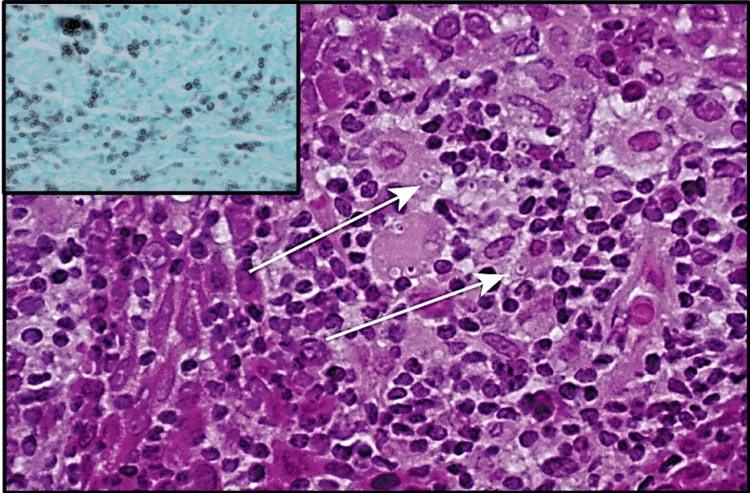
Photomicrograph revealing scattered epithelioid macrophages intermixed with lymphocytes and plasma cells. Some macrophages comprise organisms of histoplasma capsulatum (arrows) (x40). Inset: high-power photomicrograph of a tissue section readily illustrates the small yeasts of histoplasma capsulatum (Grocott-Gomori methenamine silver stain). Permission has been obtained from the original publishers to reproduce this figure from the source [23].

The lesional tissue showed either a diffuse infiltration of macrophages or, more frequently, aggregations of macrophages arranged into granulomas. Multinucleated giant cells are commonly observed in associated with the granulomatous inflammation (Figure 13). It is more difficult to identify the causative organism under the routine H&E-stained section; however, the characteristic 1- to 2-μm yeasts of *H. capsulatum* can be readily demonstrated by special stains, e.g., PAS (red-violet colored and surrounded by a light halo) and Grocott-Gomori methenamine silver (brown-black colored) methods [20,34] (Figure 13 inset).

Aspergillosis: A low-grade infection gets established in the maxillary sinus, leading to the formation of a cluster of fungal hyphae known as an aspergilloma, which may undergo dystrophic calcification, creating a radiopaque body called an antrolith within the sinus. The occurrence of aspergillosis following a tooth extraction or endodontic procedure, particularly in the posterior regions of the maxilla, is noted. Presumably, damage to the tissues makes the sinus more vulnerable to infection, resulting in symptoms such as localized pain and sensitivity, along with discharge from the nasal passages. Immunocompromised patients are at a higher risk of developing oral aspergillosis, and some researchers have proposed that the entry point could be the marginal gingiva and gingival sulcus. Initially, painful ulcerations in the gingiva are evident, followed by diffuse swelling of the surrounding mucosa and soft tissues with a gray or purplish discoloration. Without intervention, the condition may progress to extensive tissue death, manifesting as a yellow or black ulcer, accompanied by swelling of the face [20].

**Figure 14:**
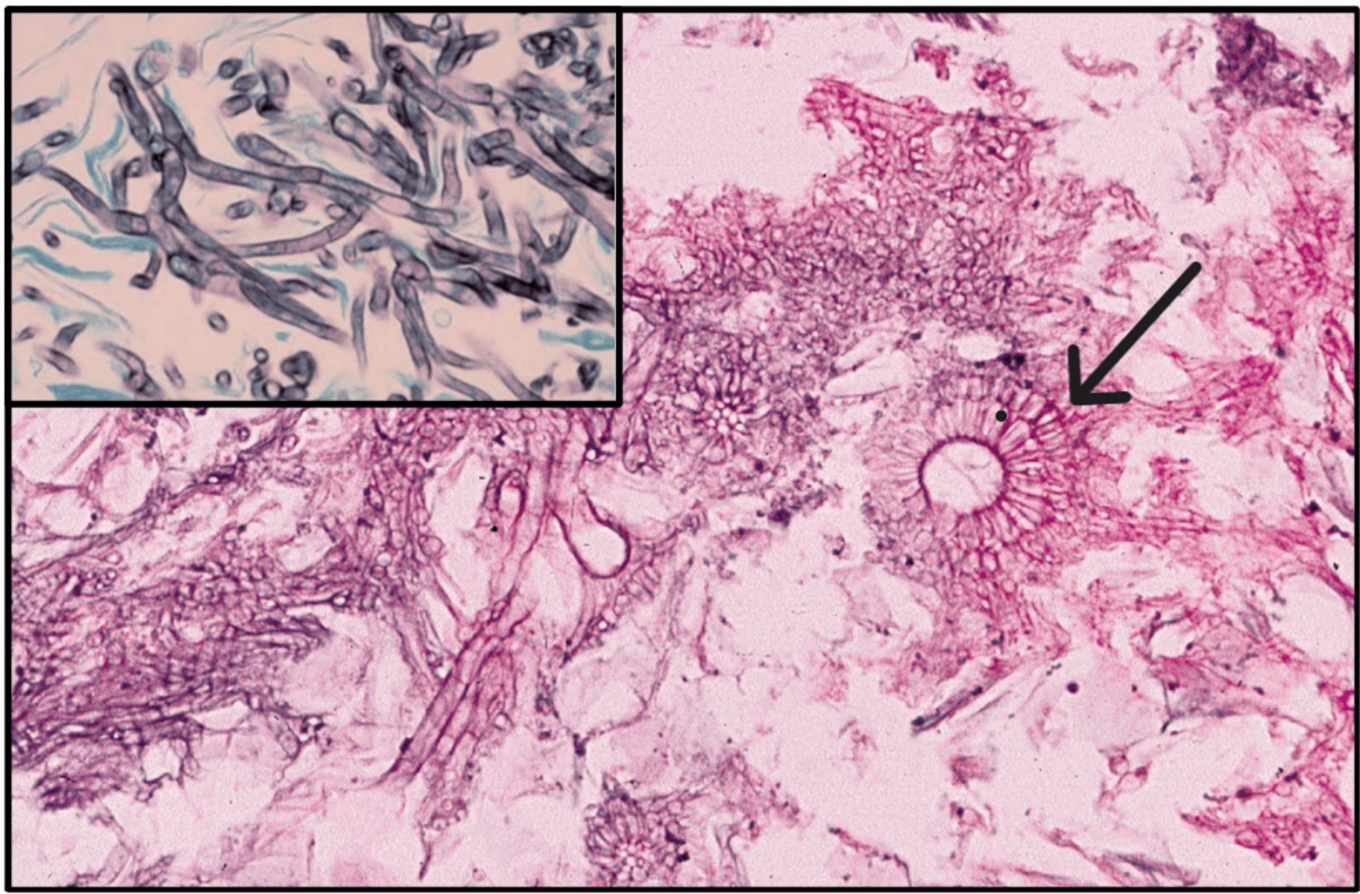
Photomicrograph shows fungal hyphae with a fruiting body (black arrow) of an Aspergillus spp. Inset: the high-power photomicrograph reveals the characteristic septate hyphae of Aspergillus spp. (Grocott-Gomori methenamine silver stain). Permission has been obtained from the original publishers to reproduce this figure from the source [20].

Tissue sections of invasive lesions caused by *Aspergillus* spp. exhibit variable numbers of branching, septate hyphae measuring about 3 to 4 μm in diameter (Figure 14). The hyphae demonstrate a propensity to bifurcate at an acute angle and infiltrate neighboring small blood vessels. Frequently, occlusion of these vessels leads to the characteristic necrotic pattern associated with this particular disease [20]. In the context of the immunocompetent individual, an inflammatory response involving granuloma formation is anticipated alongside necrosis. Conversely, in immunocompromised patients, the inflammatory response tends to be deficient or absent, resulting in significant damage to the surrounding tissue. Despite this, non-invasive manifestations of aspergillosis present distinct histopathological characteristics compared to invasive forms. For instance, an aspergilloma is typified by a complex aggregation of hyphae without any signs of tissue penetration. By contrast, allergic fungal sinusitis demonstrates extensive pools of eosinophilic inspissated mucus intermingled with layered accumulations of lymphocytes and eosinophils. The detection of fungal hyphae is relatively scanty, and even then, this identification necessitates meticulous examination following methenamine silver staining (Figure 14 inset) [20]. 

Zygomycosis/mucormycosis: Early clinical manifestations include the appearance of a reddish-black nasal turbinate and septum with a nasal discharge. The necrosis may spread to the paranasal sinuses and orbital cavities, leading to the formation of sinus tracts and sloughing of tissues. If the maxillary sinus is involved, they appear as intraoral swelling of the maxillary alveolar process, the palate, or both. If the condition is not treated, palatal ulceration may typically develop with a black and necrotic surface. Massive tissue destruction may result if the condition remains untreated [19,20].

**Figure 15:**
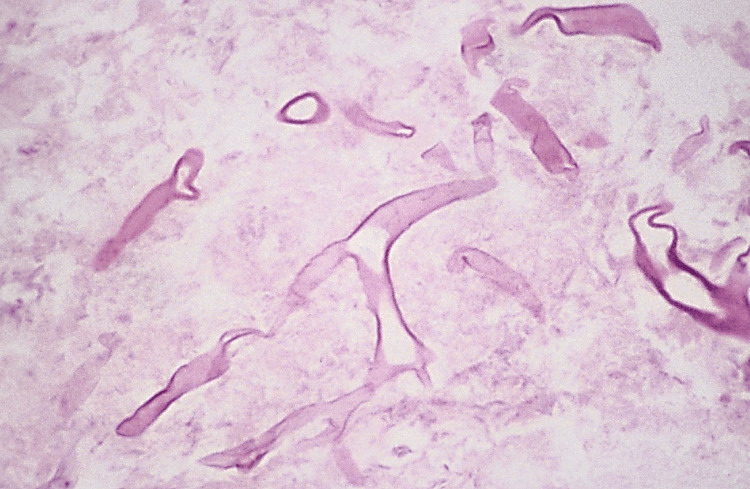
Zygomycosis: the photomicrograph demonstrates the large, nonseptate fungal hyphae distinctive of the zygomycotic organisms. Permission has been obtained from the original publishers to reproduce this figure from the source [20].

Extensive necrosis characterized by numerous large (6 to 30 μm in diameter), branching, non-septate hyphae located at the outer edge (Figure 15). These hyphae exhibit a tendency to bifurcate at right angles. The significant damage to the tissue and necrosis observed in this condition can be confidently attributed to the fungi's preference for infiltrating small blood vessels. This disrupts the normal blood circulation to the tissue, leading to infarction and necrosis. In the viable tissue, a predominance of neutrophils is typically noted, although the host's immune response to the infection may be subdued, especially in cases of immunosuppression [20].

Langerhans cell histiocytosis (LCH, histiocytosis X, Langerhans cell disease): The clinicopathological variety commonly viewed under the designation of this disease includes the following: Monostotic or polyostotic eosinophilic granuloma of bone-solitary or multiple bone lesions without visceral involvement; chronic disseminated histiocytosis, involving the bone, skin, and viscera (Hand-Schüller-Christian disease); and acute disseminated histiocytosis, prominent cutaneous, visceral, and bone marrow involvement occurring mainly in infants (Letterer-Siwe disease) [20].

Several people have overlapping clinical features that make it challenging to categorize them into one of these traditional classifications. The often-cited Hand-Schüller-Christian triad-bone lesions, exophthalmos, and diabetes insipidus are seen in a couple of patients having chronic disseminated disease. Bone lesions, either solitary or multiple, are the most common clinical presentation, which may be found in almost any bone, but the skull, ribs, vertebrae, and mandible are the most frequent sites. Lymphadenopathy usually does not have any significant visceral involvement. Jaws are mostly affected in 10-20% of all cases; dull pain and tenderness frequently coexist with bone lesions [20].

**Figure 16:**
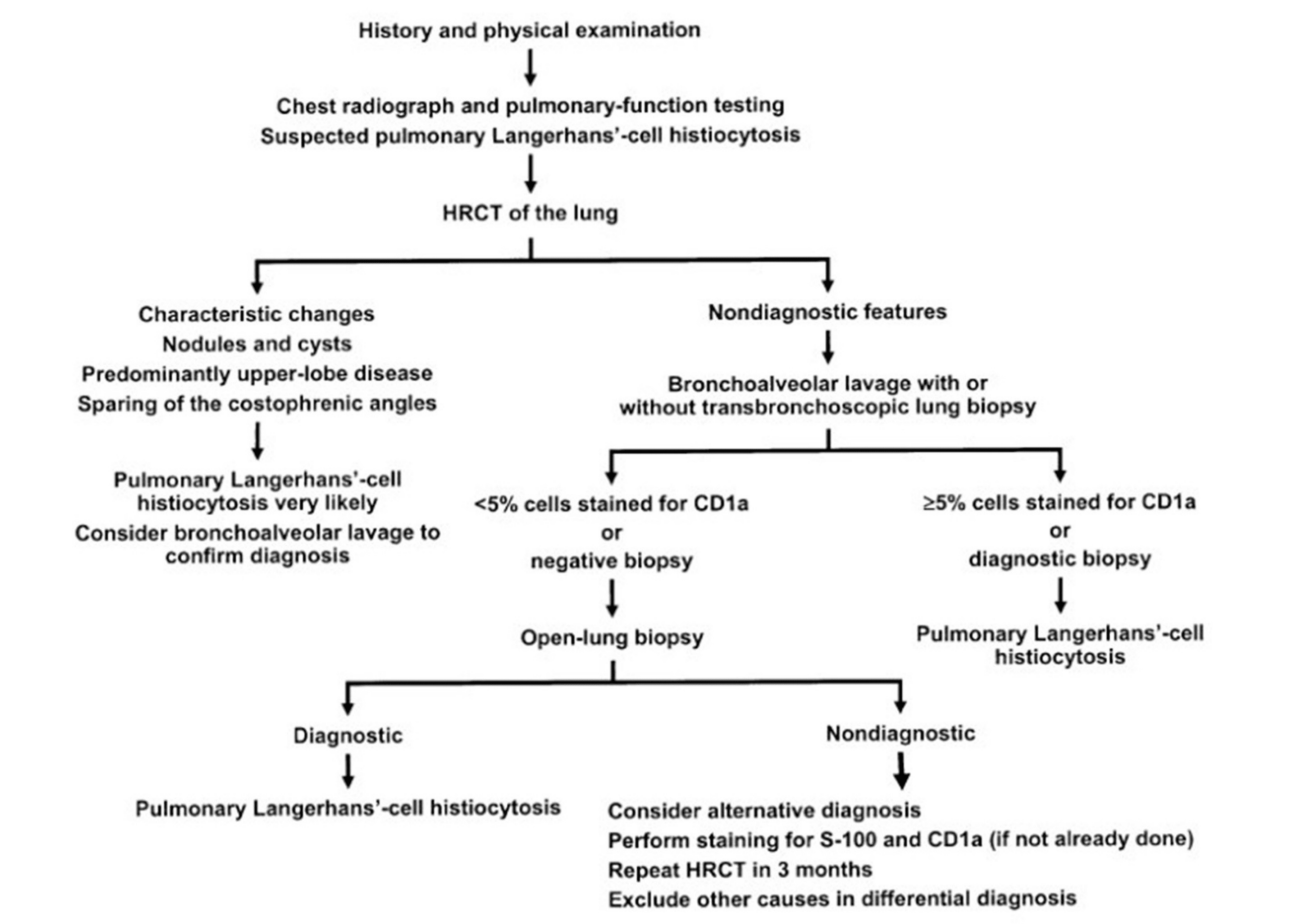
Proposed diagnostic methods for the evaluation of patients with suspicion of pulmonary Langerhan’s cell histiocytosis (PLCH). Permission has been obtained from the original publishers to reproduce this figure from the source [36].

LCH is diagnosed by histological and immunophenotypic examinations. The major markers include typical LCH cells and the presence of CD1a and/or Langerin (CD207) cells. It is no longer essential to use electron microscopy to confirm cytoplasmic Birbeck granules. A complete patient history, including smoking history, must be obtained, as well as particular physical tests, including neurological assessment of the central nervous system (CNS) and peripheral nerves (Figure 16) [35].

**Figure 17:**
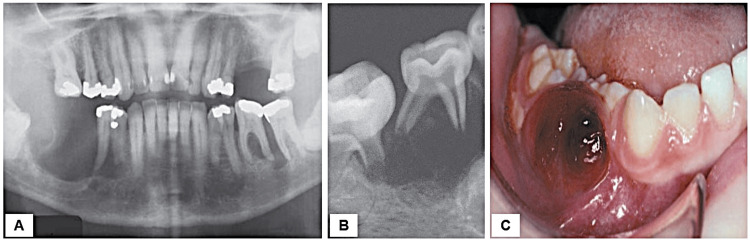
Langerhans cell histiocytosis: a) severe bone resorption involving the mandibular molar regions exhibiting similarities to advanced periodontitis; b) periapical radiograph shows marked bone resorption in the mandibular teeth in a young girl, resulting in a “floating-in-the-air” appearance; c) the lesion has extended beyond the bone producing this proliferative soft tissue mass. Permission has been obtained from the original publishers to reproduce this figure from the source [20].

In terms of radiological features, sharply defined punched-out radiolucencies lack a corticated rim; however, at times, they may present as indistinct radiolucency. Involvement of bone in the mandible typically manifests in the posterior regions, often displaying a distinctive “scooped out” aspect when the superficial alveolar bone undergoes destruction. The resultant bone damage and subsequent teeth loosening clinically bear resemblance to severe periodontitis (Figure 17A). Extensive involvement of the alveolar bone results in the teeth appearing as though they are “floating in the air” (Figure 17B). If the disease extends beyond the bone, ulcerative or proliferative mucosal lesions, as well as a proliferative gingival mass, may manifest (Figure 17C). Rarely, this progression may exclusively affect the soft tissues within the oral cavity. In addition, lesions may develop within the body of the mandible or maxilla, resembling a periapical inflammatory condition [20].

**Figure 18:**
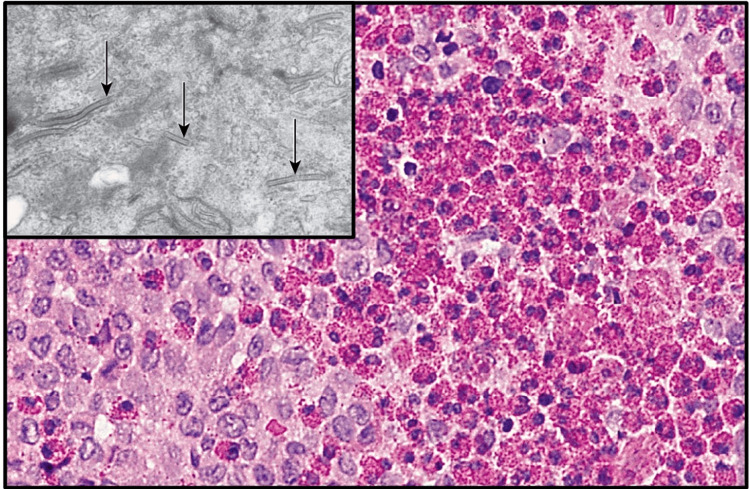
Diffuse infiltration of pale-staining Langerhans cells admixed with numerous red granular eosinophils. Inset: electron micrograph illustrating rod-shaped birbeck bodies (black arrows) in the cytoplasm of a Langerhans cell. Permission has been obtained from the original publishers to reproduce this figure from the source [20].

The histologic features of this condition are diffuse infiltration of large, pale-staining mononuclear cells that resemble histiocytes having indistinct cytoplasmic borders and rounded or indented vesicular nuclei. Variable numbers of eosinophils are commonly dispersed amidst the histiocyte-like cells, as illustrated in Figure 18. The presence of plasma cells, lymphocytes, and multinucleated giant cells is frequently observed, along with areas of necrosis and hemorrhage. Identification of lesional Langerhans cells is imperative for confirming the diagnosis, as these cells cannot be distinguished from other histiocytes through standard histologic staining methods, necessitating additional diagnostic approaches [20]. Electron microscopic assessment of the affected tissue has been considered the conventional method for numerous years due to the ultrastructural identification of Birbeck granules within Langerhans cells. These granules exhibit a rod-shaped morphology with distinctive periodicity, occasionally presenting a dilated terminal end resembling a tennis racket appearance. Their intracellular localization can be discerned by electron microscopy, facilitating their discrimination from other mononuclear phagocytes (Figure 18 (inset)). Nowadays, many laboratories opt for immunohistochemical techniques to detect the Langerhans cells within the lesion, primarily relying on the immunoreactivity displayed toward antibodies targeting CD-1a or CD-207. The latter marker, specifically CD-207, demonstrates a higher specificity for Langerhans cells. Furthermore, the lesional cells exhibit a certain degree of S-100 protein immunoreactivity, along with a potential inclination toward peanut agglutinin (PNA) [20,37].

Sarcoidosis: The clinical features and common clinical symptoms of this condition include dyspnea, dry cough, chest pain, fever, malaise, fatigue, arthralgia, and weight loss. Approximately 20% of patients have no symptoms, and the disease is discovered on routine chest radiographs and involves multiple organs, the lungs being the most frequently affected site, followed by the lymph nodes, skin, eyes, salivary glands, lymphoid tissue, bone, joints, muscle, heart, nerve, liver, and kidney. The mediastinal and paratracheal lymph nodes are commonly involved and chest radiographs reveal bilateral hilar lymphadenopathy. The cutaneous manifestations are erythema nodosum, symmetrical, elevated, indurated, purplish plaques, pigmentations, or ulcers. The ocular lesions are anterior uveitis, lesions of the conjunctiva and retina and keratoconjunctivitis sicca, xerostomia, and significant enlargement of any major or minor salivary gland. The salivary gland enlargement, xerostomia, and keratoconjunctivitis sicca can combine to mimic Sjögren syndrome. Two distinctive clinical syndromes are associated with acute sarcoidosis. Löfgren’s syndrome consists of erythema nodosum, bilateral hilar lymphadenopathy, and arthralgia. Patients with Heerfordt’s syndrome (uveoparotid fever) have parotid enlargement, anterior uveitis of the eye, facial paralysis, and fever. Oral manifestations in sarcoidosis are very rare. They appear as asymptomatic, well-circumscribed submucosal mass, isolated papule, area of granularity, or ulceration. The mucosal lesions might be normal in color, brown-red, violaceous, or hyperkeratotic. The most frequently affected areas are the buccal mucosa, followed by the gingiva, lips, floor of mouth, tongue, and palate. Most cases appearing on the floor of the mouth involve salivary glands and create mucus extravasation. Intraosseous lesions affect either jaw and appear as ill-defined radiolucencies that occasionally erode the cortex but never create expansion [20].

**Figure 19:**
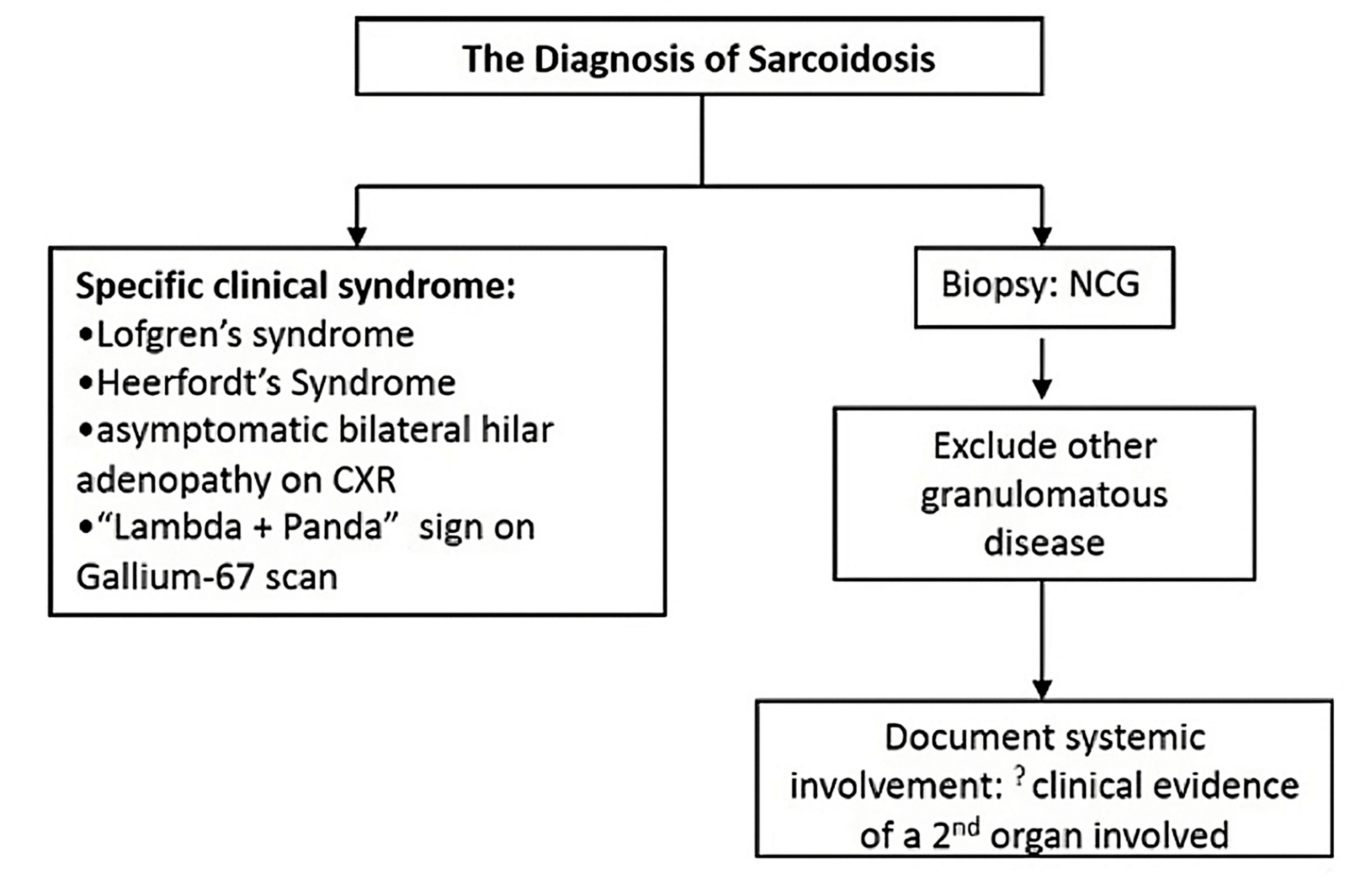
Diagnostic methods for sarcoidosis. Permission has been obtained from the original publishers to reproduce this figure from the source [38].

The diagnosis of sarcoidosis can be established without the need for a tissue biopsy, as depicted on the left side of the illustration (Figure 19). This necessitates the presence of specific clinical manifestations that exhibit a high level of specificity for the diagnosis, as outlined in one of the conditions within the box on the left side of the illustration. In cases where none of these distinct clinical presentations manifest, confirmation of the diagnosis necessitates tissue biopsy to establish the presence of granulomatous inflammation, ruling out other potential causes for such inflammation, and providing evidence that the disease is systemic, thereby indicating its presence beyond a single organ (pathway on the right side of the figure) (Figure 19) [38].

**Figure 20:**
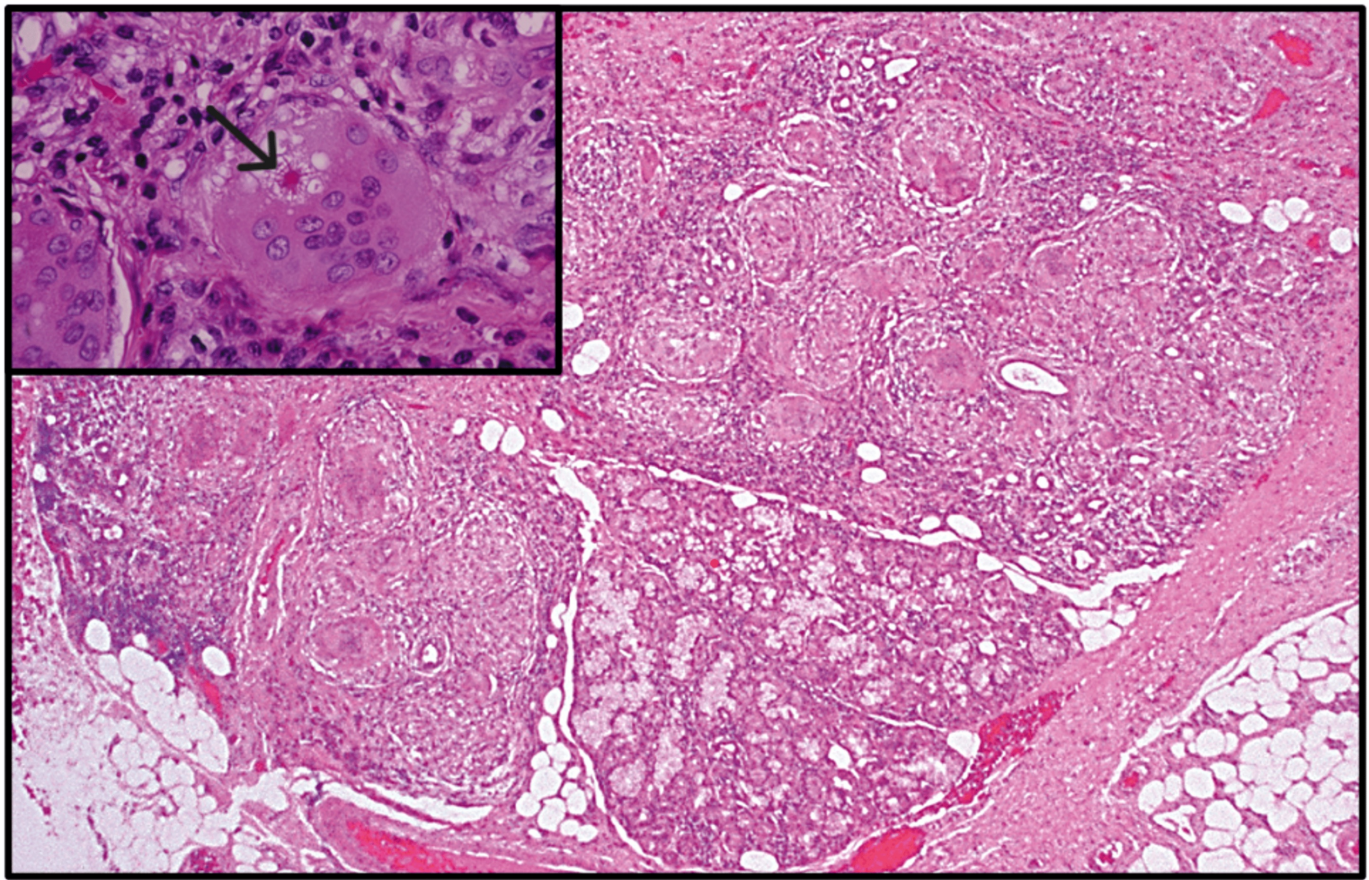
Sarcoidosis: photomicrograph of a labial minor salivary gland showing granulomatous inflammation distinguised by encircled aggregates of histiocytes, lymphocytes, and multinucleated giant cells. Inset: photomicrograph revealing a multinucleated giant cell with an intracytoplasmic asteroid body (black arrow). Permission has been obtained from the original publishers to reproduce this figure from the source [20].

In terms of the histologic features, a microscopic analysis of sarcoidosis reveals a characteristic presentation of granulomatous inflammation. Aggregates of epithelioid histiocytes are tightly grouped together, accompanied by a periphery of lymphocytes. Langhans' or foreign-body-type giant cells are dispersed among the histiocytes (Figure 20). The granulomas frequently exhibit laminated basophilic calcifications, referred to as Schaumann bodies (resulting from lysosome degeneration), as well as stellate inclusions named asteroid bodies (comprising trapped collagen fragments) (Figure 20 inset) [20,39].

The diagnosis is determined through the examination of clinical and radiographic manifestations, histopathological characteristics, and the absence of positive results in special stains and cultures for microorganisms. Elevated levels of angiotensin-converting enzyme (ACE) in the serum, along with a thorough documentation of pulmonary manifestations, provide substantial backing for the diagnosis. Nevertheless, it should be noted that elevated ACE levels are detected in just 60% of individuals with sarcoidosis and in a minority of those with oral manifestations. Other laboratory abnormalities include eosinophilia, leukopenia, anemia, thrombocytopenia, and elevation of the serum alkaline phosphatase level, erythrocyte sedimentation rate, serum calcium concentration, and urinary calcium level [20,40].

Crohn’s disease: For the oral manifestations of this condition, the buccal mucosa is frequently affected, demonstrating lesions with a cobblestone-like appearance. Similarly, the vestibule may exhibit linear and hyperplastic folds resembling denture-induced hyperplasia. The lips can show diffuse swelling and induration, while the gingiva and alveolar mucosa may present with a granular erythematous swelling. In addition, the palate may develop multiple ulcers. Glossitis might manifest as a result of vitamin B12 malabsorption. Oral lesions can manifest before or after intestinal lesions, and like the latter, they often display periods of inactivity interspersed with exacerbations of the condition [19].

**Figure 21:**
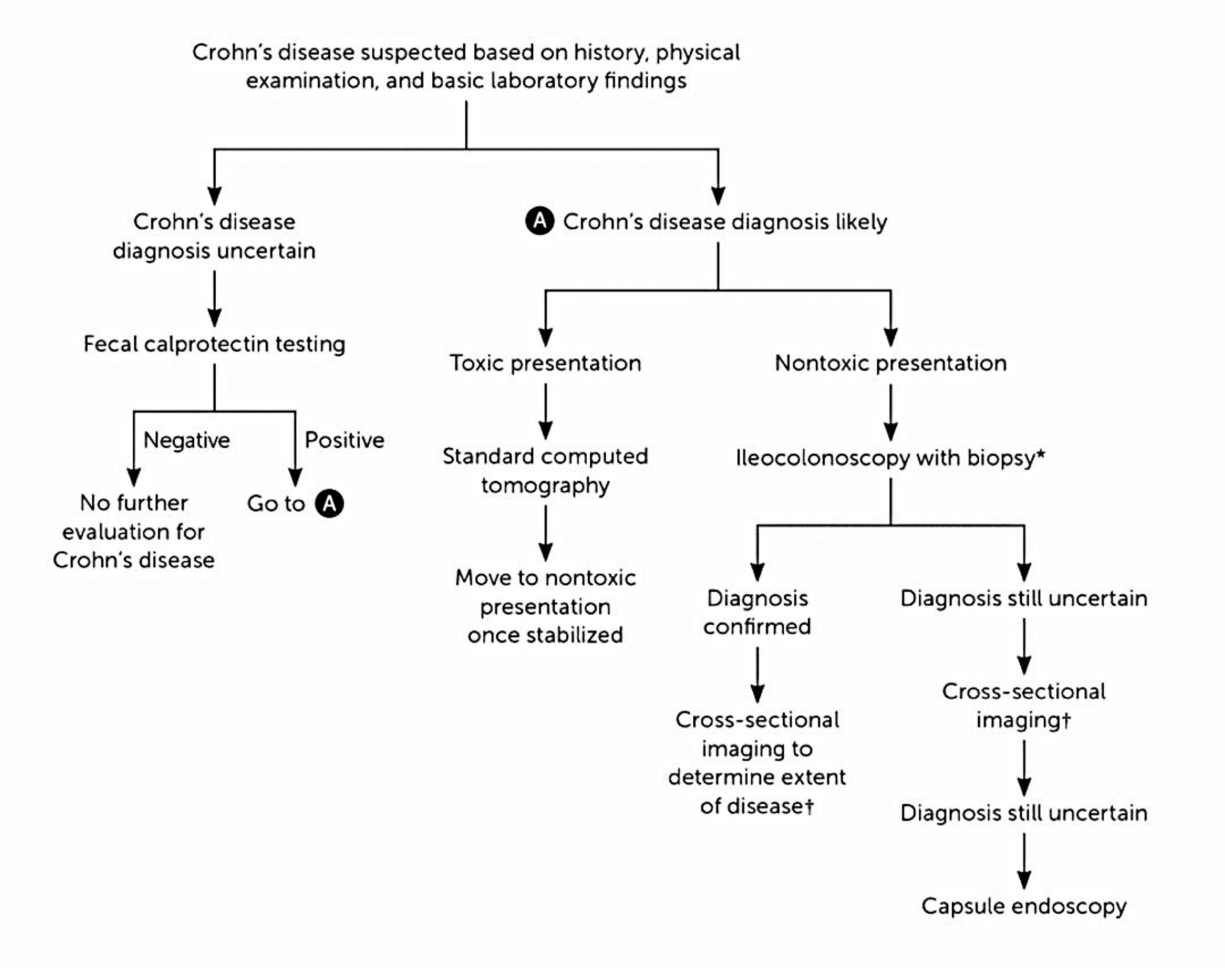
Algorithm for diagnosing Crohn's disease. * esophagogastroduodenoscopy may be deemed, especially for children and/or in the presence of upper gastrointestinal symptoms; † choice of cross-sectional imaging technique depends on several factors. Permission has been obtained from the original publishers to reproduce this figure from the source [41].

The identification of Crohn's disease is established through a combination of clinical observations along with endoscopic, histologic, radiologic, and/or biochemical examinations. The determination to proceed with the diagnosis is primarily guided by the patient's medical history, physical assessment, and fundamental laboratory results. In cases where both ileocolonoscopy and cross-sectional imaging yield no conclusive evidence, yet suspicions of Crohn's disease persist, capsule endoscopy becomes the subsequent investigative measure. In the event of a negative outcome from this procedure, it can be reasonably inferred that the disease is absent (Figure 21) [41].

**Figure 22:**
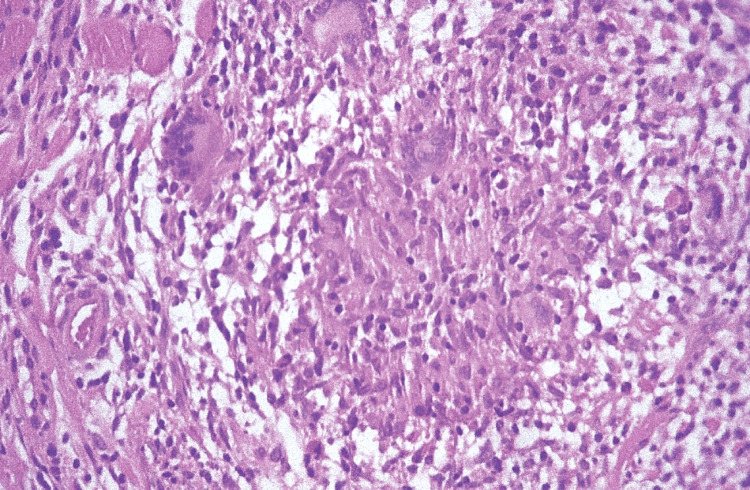
Crohn’s disease: medium-power photomicrograph of an oral lesion showing a non-necrotizing granuloma in the submucosal connective tissue. Permission has been obtained from the original publishers to reproduce this figure from the source [20].

In terms of histologic features, the lesional tissue procured from the intestine or the oral mucosa reveals non-necrotizing granulomatous inflammation in the submucosal connective tissue (Figure 22). The intensity of the granulomatous inflammation can exhibit significant variation among patients and different locations within the same patient. Consequently, a negative biopsy outcome at any specific site and time may not definitively exclude a diagnosis of Crohn’s disease. Analogous to the clinical manifestations, the histopathological presentation is rather nonspecific, resembling orofacial granulomatosis. This presentation comprises fibrosis and a focal dense aggregation of lymphocytes and plasma cells, with dilated lymph vessels. The identification of noncaseating granulomas, typically small and composed of macrophages, epithelioid cells, and sporadic giant cells, is common [20].

## Conclusions

Granulomatous diseases are a heterogeneous group of chronic inflammatory disorders whose pathogenesis is triggered by an array of infectious and non-infectious agents. Granulomatous diseases of the oral tissues are uncommon and are difficult to diagnose due to various etiological agents and may be localized or a manifestation of systemic and disseminated disease and often present in the oral soft tissues with nonspecific signs and symptoms. Oral granulomatous diseases are a large group of lesions that share a similar histogenesis of granuloma formation. Therefore, an extensive clinical, microscopic, and laboratory evaluation is required to identify the source of oral granulomatous diseases. The comprehensive knowledge about granulomatous diseases and their diversity presents considerable diagnostic and management challenges to pathologists or surgeons and physicians in general. Herewith, a humble comprehensive review of literature on granulomatous lesions of the oral cavity has been put forth, which may serve as a guiding light, for undergraduate and post-graduate medical and/or dental fraternity along with researchers in their pursuit of knowledge and clarity and in turn improving their overall conceptualization and research methodology on the same.
